# Genome-Wide SNP Analysis Reveals the Population Structure and the Conservation Status of 23 Italian Chicken Breeds

**DOI:** 10.3390/ani10081441

**Published:** 2020-08-18

**Authors:** Filippo Cendron, Francesco Perini, Salvatore Mastrangelo, Marco Tolone, Andrea Criscione, Salvatore Bordonaro, Nicolaia Iaffaldano, Cesare Castellini, Margherita Marzoni, Arianna Buccioni, Dominga Soglia, Achille Schiavone, Silvia Cerolini, Emiliano Lasagna, Martino Cassandro

**Affiliations:** 1 Department of Agronomy, Food, Natural Resources, Animals and Environment, University of Padova, Viale dell’Università, 16, 35020 Legnaro, Italy; filippo.cendron@unipd.it (F.C.); martino.cassandro@unipd.it (M.C.); 2 Department of Agricultural, Food and Environmental Sciences, University of Perugia, Borgo XX Giugno, 74, 06121 Perugia, Italy; francesco.perini@studenti.unipg.it (F.P.); cesare.castellini@unipg.it (C.C.); 3 Department of Agricultural, Food and Forest sciences, University of Palermo, Viale delle Scienze, Ed. 4, 90128 Palermo, Italy; salvatore.mastrangelo@unipa.it (S.M.); marco.tolone@unipa.it (M.T.); 4 Department of Agronomy, Food, and Environment, University of Catania, Via Valdisavoia, 5, 95100 Catania, Italy; a.criscione@unict.it (A.C.); s.bordonaro@unict.it (S.B.); 5 Department of Agricultural, Environment and Food, University of Molise, Via De Sanctis s/n, 86100 Campobasso, Italy; nicolaia@unimol.it; 6 Department of Veterinary Science, University of Pisa, Viale delle Piagge 2, 56124 Pisa, Italy; margherita.marzoni@unipi.it; 7 Department of Agriculture, Food, Environment and Forestry, University of Firenze, Via di San Bonaventura, 50145 Firenze, Italy; arianna.buccioni@unifi.it; 8 Department of Veterinary Science, University of Torino, Largo Paolo Braccini, 2, 10095 Grugliasco, Italy; dominga.soglia@unito.it (D.S.); achille.schiavone@unito.it (A.S.); 9 Department of Veterinary Science, University of Milano, Via Trentacoste, 2, 20134 Milano, Italy; silvia.cerolini@unimi.it

**Keywords:** biodiversity, local breeds, genetic diversity, safeguard, poultry

## Abstract

**Simple Summary:**

To assess the conservation status and the genomic variability of Italian chicken breeds, we carried out genome-wide analyses using single nucleotide polymorphism (SNP) molecular markers. These results increase knowledge and can aid with the development of conservation plans for local Italian chicken breeds to safeguard their genetic variability.

**Abstract:**

The genomic variability of local Italian chicken breeds, which were monitored under a conservation plan, was studied using single nucleotide polymorphisms (SNPs) to understand their genetic diversity and population structure. A total of 582 samples from 23 local breeds and four commercial stocks were genotyped using the Affymetrix 600 K Chicken SNP Array. In general, the levels of genetic diversity, investigated through different approaches, were lowest in the local chicken breeds compared to those in the commercial stocks. The level of genomic inbreeding, based on runs of homozygosity (F_ROH_), was markedly different among the breeds and ranged from 0.121 (Valdarnese) to 0.607 (Siciliana). In all breeds, short runs of homozygosity (ROH) (<4 Mb in length) were more frequent than long segments. The patterns of genetic differentiation, model-based clustering, and neighbor networks showed that most breeds formed non-overlapping clusters and were clearly separate populations, which indicated the presence of gene flow, especially among breeds that originated from the same geographical area. Four genomic regions were identified as hotspots of autozygosity (islands) among the breeds, where the candidate genes are involved in morphological traits, such as body weight and feed conversion ratio. We conclude that the investigated breeds have conserved authentic genetic patterns, and these results can improve conservation strategies; moreover, the conservation of local breeds may play an important role in the local economy as a source of high-quality products for consumers.

## 1. Introduction

The poultry industry represents an essential and constantly developing branch of agriculture production as an important food provider. Unfortunately, the poultry sector has undergone significant loss in terms of animal genetic resources and the erosion of many genotypes due to replacement by higher-performing commercial hybrids or indiscriminate crossbreeding, related to highly efficient selection programs [1]. Thus, intensive breeding has led to important changes in the patterns of the genomic diversity and compromised the consideration and the survival of local chicken breeds. Worldwide data show that about 50% of known breeds of poultry are classified as extinct, critical, or endangered, and local breeds are often subjugated by cosmopolitan chicken breeds [2]. It is therefore important to preserve genetic resources that may help to meet future demands in animal breeding. Local chicken breeds are recognized as livestock populations that should be conserved and that represent an important source of novel genetic diversity for microevolution and environment adaptability [3]. To support the conservation of local breeds, several actions have been planned over the years (i.e., a conservation scheme, breeding plans, and breeding niches), supported by genetic studies [4,5,6].

In Italy, the interest in the conservation of local poultry breeds began about 20 years ago with regional plans for conservation, followed recently by national involvement [2,5]. From this background, the project TuBAvI—“Conservation of biodiversity in Italian poultry breeds” (https://www.pollitaliani.it/en/project/)—was developed to evaluate the safeguarding, conservation, and improvement of the Italian poultry genetic resources, represented by many autochthonous breeds that were historically present in the country.

Due to the recent advent of high-throughput affordable genotyping techniques, the fine genome-wide analysis of the genetic structure and relationships between chicken populations has become possible [7]. These technologies have provided new perspectives on livestock genetics with the introduction of genomic approaches in conservation programs for small and endangered populations [8]. A number of the considered breeds in the TuBAvI project have already been analyzed with molecular markers [5,6,9]; however, due to a lack of population genomic studies, it was not possible to make inferences about the impact of genetic traits on whole-genome variation.

Thus, in this work, we aimed to provide a high-resolution genetic overview of the genome-wide diversity and population structure of 23 Italian local breeds and four commercial hybrids. Most of the reported breeds are officially recognized by the Italian authorities and by the Food and Agriculture Organization of the United Nations (FAO). Only the Cornuta di Caltanissetta and Valplatani breeds are not yet recognized. We investigated the level of genetic diversity, population structure, admixture patterns, and relationships among these Italian chicken breeds, in order to verify the effectiveness of the conservation programs.

## 2. Materials and Methods

### 2.1. Samples and Genotyping

Blood samples and animal care were performed in compliance with the European rules (Council Regulation (EC) No. 1/2005 and Council Regulation (EC) No. 1099/2009). The animals identified in this study were intended for slaughter at the end of their production cycle, and samples were collected by official veterinarians who adhered to standard procedures for animals for slaughter.

A total of 582 samples were collected from 20 to 24 animals (half males and females, unrelated) per breed. We sampled 23 different local chicken breeds (Ancona (ANC), Bianca di Saluzzo (BSA), Bionda Piemontese (BPT), Cornuta di Caltanissetta (COR), Livorno Bianca (PLB), Livorno Nera (PLN), Mericanel della Brianza (MER), Modenese (MOD), Mugellese (MUG), Ermellinata di Rovigo (PER), Millefiori di Lonigo (PML), Padovana Argentata (PPA), Polverara Bianca (PPB), Padovana Camosciata (PPC), Padovana Dorata (PPD), Polverara Nera (PPN), Pepoi (PPP), Robusta Lionata (PRL), Robusta Maculata (PRM), Romagnola (ROM), Siciliana (SIC), Valdarnese (VLD), and Valplatani (VLP)). For the four commercial stocks (Broiler Ross 708 (708), Eureka (EUK), Hy-lyne white eggs (HYL), and ISA Brown (ISA)), 9 to 13 animals per breed were included. All of the blood samples (about 2 mL) were collected from ulnar veins and stored in Vacutainers^®^ tubes containing EDTA as an anticoagulant. All the studied breeds were collected from at least three different farms (except for VLP and COR, due to their small population sizes) to obtain a representative sampling of the breeds.

DNA extraction and genotyping were performed at Neogen (Ayr, Scotland, UK) using a commercial kit and the Affymetrix Axiom 600 K Chicken Genotyping Array, containing 580,961 SNPs. The Gallus_gallus-5.0 chicken assembly was used in this study as the reference genome [10], with markers positioned on chromosomes between 1 and 28 included. The raw dataset was filtered by the following parameters: (i) SNPs with a call rate < 95% and (ii) minor allele frequency < 5%, and (iii) animals with more than 10% of missing genotypes were removed. Files were edited using PLINK 1.9 [11].

PLINK 1.9 [11] was also used to estimate the average minor allele frequency (MAF) (≥0.05), observed (H_o_) and expected (H_e_) heterozygosity, and genomic inbreeding, which is based on the difference between the observed and expected numbers of homozygous genotypes (*F*_HOM_).

### 2.2. Admixture and Genetic Relationship

The population structure was investigated by applying the model-based clustering algorithm run in ADMIXTURE from *K* = 2 to 30 [12]. The cross-validation procedure was used to estimate the most likely number of populations; the K value that minimizes the cross-validation prediction error was then assumed as the most likely. The BITE R package was used to graphically represent the results [13]. Pair-wise genetic relationships within and between the breeds were estimated using a matrix of genome-wide identity-by-state (IBS) genetic distances on PLINK 1.9 [11] and plotted using a multidimensional scaling (MDS) plot that represented the components C1 and C2.

Phylogenetic relationships among the breeds were analyzed by determining the Reynolds genetic distances using the R studio package *ape* [14]. Neighbor networks were constructed from the estimated genetic distances using SplitsTree [15]. A neighbor-joining tree was also constructed based on individual allele-sharing distances (--distance 1-IBS in PLINK) and visualized using FigTree [15]. Graphical representation was created using the statistical R software [16].

### 2.3. Runs of Homozygosity

Assessment of the runs of homozygosity (ROH) was performed for each animal using PLINK 1.9 [11]. To define ROH, the following parameters were fixed: (1) the minimum length was set to 1 Mb *(--homozig-kb*), (2) two missing SNPs and up to one possible heterozygous genotype were allowed in the ROH (*--homozyg-window-missing 2* and *--homozyg-window-het 1*), (3) the minimum number of SNPs that constituted the ROH was set to 100 (*[--homozyg-snp 100*), (4) the minimum SNP density per ROH was set to one SNP every 100 kb (*--homozyg-density 100*), and (5) the maximum gap between consecutive homozygous SNPs was 1000 kb (*[--homozyg-gap 1000*). The total length of the genome covered by ROH was divided by the total chicken autosomal genome length covered by the SNP array (944,270 kb) to evaluate the individual genomic inbreeding coefficient using the ROH data (*F*_ROH_) [17]. Each ROH was clustered on its physical length in seven groups: 1 to ≤5, 5 to ≤10, 10 to ≤15, 15 to ≤20, 20 to ≤25, 25 to ≤30, and >30 Mb. The mean sum of the ROH per breed was calculated for each length group by summing all the ROH values per animal in that category and averaging per breed. The total percentage of SNPs clustering inside ROH was determined by counting the number of times that each target appeared in a ROH and dividing this by the total number of animals (582). To identify regions of high homozygosity, called ROH islands, the top 0.999% of SNPs in the locus homozygosity range were selected. Subsequently, the annotation of gene mapping within ROH islands was also conducted using the list of the chicken autosome *Gallus_gallus 5.0* from the Ensembl database (http://www.ensembl.org). To clarify the gene identity, the quantitative trait loci (QTL) database (https://www.animalgenome.org/cgi-bin/QTLdb/GG/index) was interrogated for the presence of QTLs in the ROH islands. To investigate the biological function and the phenotypes that are known to be affected by each annotated gene, a comprehensive search in the available literature was conducted.

## 3. Results

**Figure 1:**
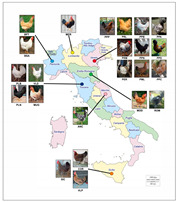
Schematic representation of chicken breed origins in Italy (figure taken from http://www.d-maps.com and adapted for illustrative purposes only). For a full definition of breeds, see Table 1.

A total of 23 local chicken breeds spread throughout Italy were studied (Figure 1). The final number of animals and SNPs after filtering were 582 and 474,412 (starting from 588,900), respectively. All of the animals included in the analysis had high quality genotyping.

### 3.1. Analysis of Whole-Genome Diversity

The genetic diversity parameters are shown in Table 1. The MAF value is approximately uniform among the local breeds, ranging from 0.241 ± 0.331 (PPA) to 0.309 ± 0.321 (PER). He and Ho had higher oscillation, reaching their maximum in BSA (0.336 ± 0.151 and 0.339 ± 0.172, respectively). The minimum value was observed in the Siciliana breed, both for He (0.123 ± 0.189) and Ho (0.129 ± 0.205). The average *F_HOM_* showed large differences among breeds, ranging from 0.076 ± 0.059 (BSA) to 0.648 ± 0.034 (SIC). All the commercial breeds showed negative values of *F_HOM_* (ISA = −0.028 ± 0.017; HYL = −0.020 ± 0.008; EUK = −0.018 ± 0.013; 708 = −0.005 ± 0.009), associated with higher He and Ho (Table 1).

### 3.2. Analysis of Genetic Distance and Population Structure

**Figure 2:**
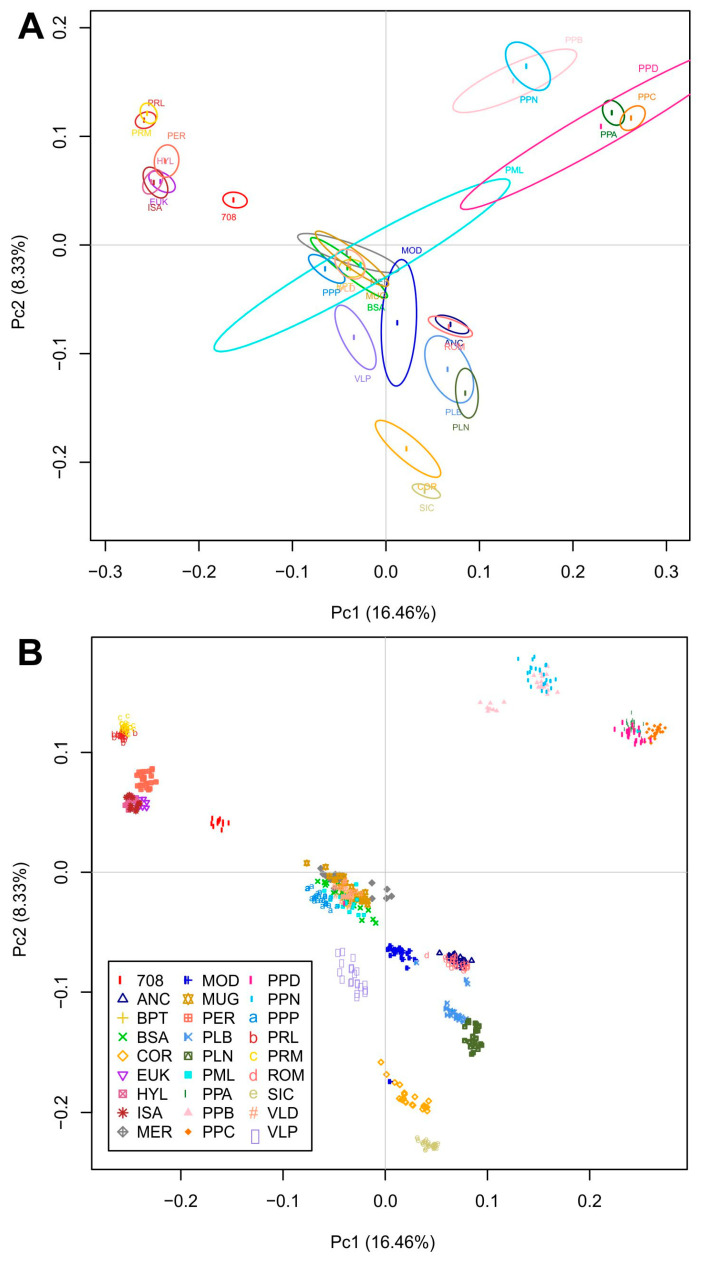
Genetic relationships among the 27 chicken breeds in this study as inferred by multidimensional scaling (MDS) analysis using (**A**) the breed-average coordinates of eigenvalues of C1 and C2 and (**B**) all of the individuals per breed. Breed acronyms are reported in Table 1.

The MDS plot in Figure 2 represents the genetic relationship among the Italian local and commercial breeds, with the breed-average coordinates of eigenvalues of C1 and C2 plotted. Several breeds created an overlapping cluster, with exceptions for some separate groups (Figure 2B). The component C2 allowed us to separate the major central cluster from the other clusters in the top left and top right of the MDS. The C1 component more clearly separates the clusters in the top right, highlighting two distinct subgroups. Thus, we can identify three major groups: the first in the top left representing the commercial breeds and PER, PRL, and PRM; the second in the top right quarter that includes the populations belonging to the Padovana and Polverara breeds; and the third, with all other breeds, in the center of the MDS, with another small group at the bottom that includes COR and SIC (Figure 2A). With regard to the top left group, the 708 Broiler breed is more detached than the other commercial breeds, and the PRL and PRM breeds form a sub-cluster. In the top right group, C1 and C2 underlined a subgroup composed of the Polverara and Padovana breeds as expected. The PPB and PPN breeds could be identified as separate clusters, except for some individuals of PPB that fall within the PPN group (Figure 2B).

**Figure 3:**
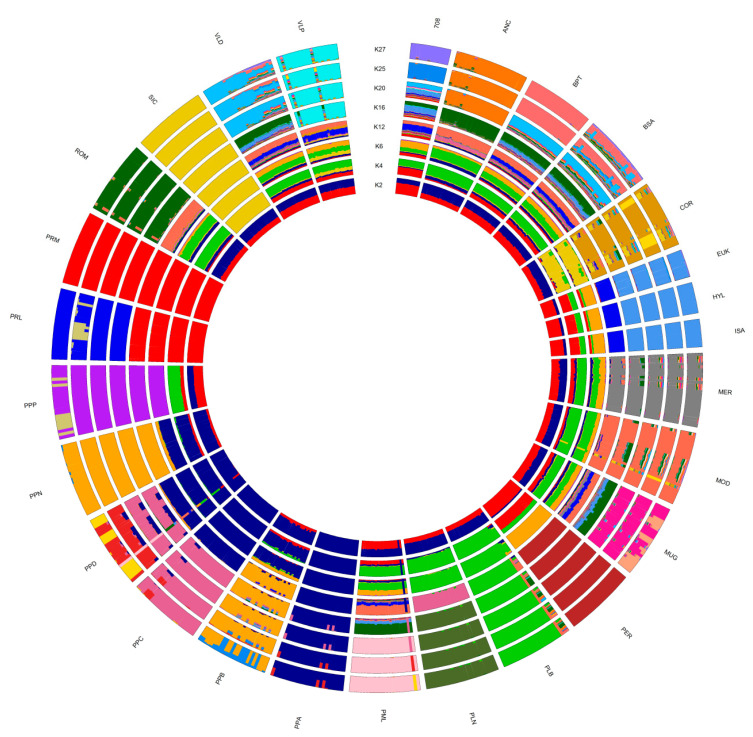
Maximum likelihood estimation calculated with the admixture algorithm. The inferred clusters (*K*) were represented from *K* = 2 to 27. Breed acronyms are reported in Table 1.

The population structure inferred by using ADMIXTURE (Figure 3), considering a range from 2 to 30 potential clusters (*K*), showed that the best fitting number of populations present in the total sample was *K* = 25. As shown by the C1 and C2 components of the MDS plot, the inferred breed structure at *K* = 2 distinguishes PRM and PRL (red), as well as PPA, PPC, and PPD (blue). PLB (green) as well as the COR and SIC breeds were separated from the others (ochre color) at *K* = 4. At *K* = 6, the PPP and PER breeds reached their genetic identity, followed by MOD, MER, PLN, and the three commercial breeds (EUK, HYL, and ISA) at *K* = 12. The ROM and ANC breeds exhibited the same genetic structure at *K* = 16; at this *K* value, a breed-specific cluster was also observed for VLP. When *K* increased from 16 to 27, the breeds were progressively assigned to separate clusters: PML, MUG, and VLD breeds at *K* = 20, and 708 Broiler and BPT at *K* = 25, whereas BSA never reached a genetic identity; at *K* = 27 its genetic background is not clear. Notably, EUK, HYL, and ISA shared ancestral genetic components from *K* = 2 to 27.

**Figure 4:**
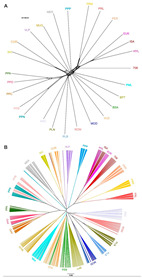
Neighbor-joining tree constructed on the Reynold’s genetic distance for the breeds considered (**A**) and based on individual allele-sharing distances (**B**). Breed acronyms are reported in Table 1.

To provide additional insight into the relationships and patterns of divergence, we constructed a neighbor-net based on Reynolds genetic distances (Figure 4A). Consistent with the MDS plot, the neighbor-net shows several clear cluster relationships between breeds, including the Padovana and Polverara breeds, and the commercial stocks with Ermellinata di Rovigo, Robusta Lionata, and Maculata breeds. The neighbor joining (NJ) tree based on allele sharing distance (ASD) separated individuals according to their population of origin (Figure 4B). Within all the breeds, the genetic distance of the individual animals set up several sub-clusters related to the breeding of origin, for instance, in the clusters of PPP, PRL, and PER (Figure 4B). Finally, some samples seemed to be misclassified for breed, probably due to sampling error.

### 3.3. Run of Homozygosity Analysis

**Figure 5:**
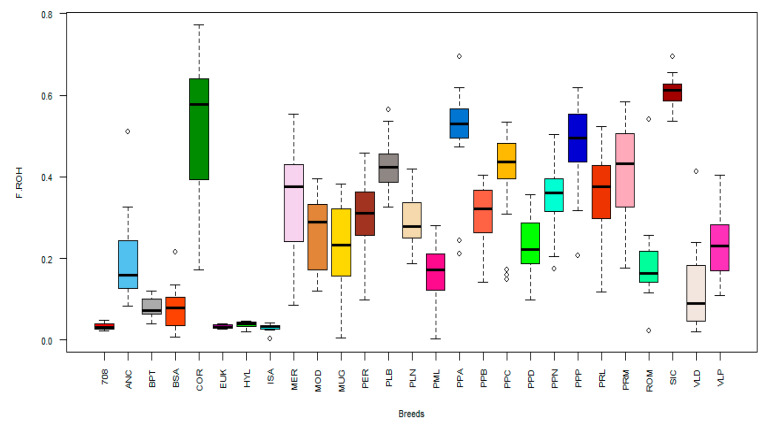
Boxplot of the inbreeding coefficient (F_ROH_) estimated from runs of homozygosity for each breed considered in this study.

The genomic inbreeding of individuals was also investigated using ROH data. The distributions of F_ROH_ for each breed are reported in Figure 5, and the mean values are given in Table 2. The SIC breed showed the highest value (F_ROH_ = 0.607 ± 0.037), whereas the BSA and BTP breeds showed the lowest values (F_ROH_ = 0.081 ± 0.057 and 0.081 ± 0.024, respectively). The commercial stocks showed the lowest mean values of inbreeding (708 = 0.034 ± 0.009; EUK = 0.033 ± 0.005; ISA = 0.030 ± 0.011; HYL = 0.038 ± 0.008) (Table 2). In general, medium F_ROH_ values were found for the other breeds.

The mean ROH segments per individual ranged from 157.58 (PRM) to 16.22 (ISA). The ROH values were clustered by their physical length into seven categories, and the mean sum of ROH values per breed was evaluated (Figure S1). Breeds differ in terms of ROH length categories. The histogram showed that for all the breeds, except for commercial stocks, the majority of the segments are less than 5 Mb in length. The PPP, PRL, and PRM breeds have a larger mean portion of their genome (79.51, 78.46, and 86.14 Mb, respectively) covered in shorter ROH (1–5 Mb), in agreement with the M_ROH_ results. COR, PPA, SIC, and VLP have a large mean portion of their genome covered in longer ROH >30 Mb.

**Figure 6:**
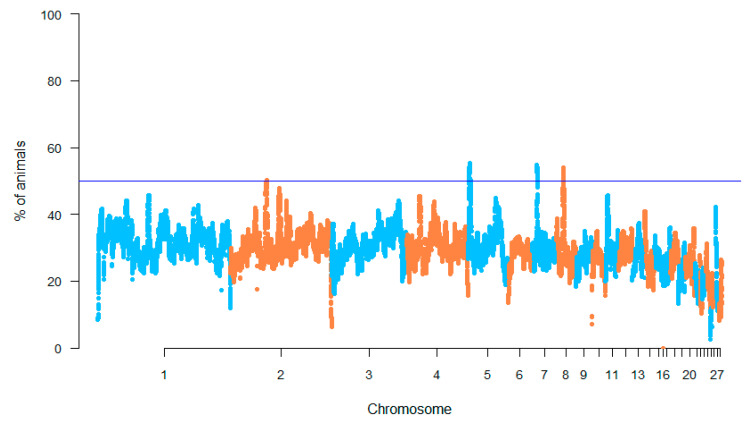
Manhattan plot of each single nucleotide polymorphism (SNP) significance in runs of homozygosity. Blue line indicates the top 0.999% of SNPs.

The top 0.999% of the SNPs in the locus homozygosity range were considered to identify the genomic regions that were most commonly associated with ROH in the studied breeds, as an indicator of a possible ROH hotspot in the genome (Figure 6). The chromosome position, number of SNPs, and start and end of these regions with the annotated genes are reported in Table 3. Four regions were identified: on chromosome 2 (53.13–53.20 Mb), chromosome 5 (2.12–3.73 Mb), chromosome 7 (6.77–7.89 Mb), and chromosome 8 (9.51–10.60 Mb). Two genes were identified on chromosome 2—*TPK1* and *LOC107051643*—whereas on chromosomes 5, 7, and 8, several annotated genes were found. In addition, located on chromosome (LOC) genes were found inside all of the ROH islands; however, their functions are still unknown (Table 3). To complete the genetic profile, the QTL database was interrogated. Several QTLs have been reported on these genomic regions in chickens. As reported in Table 3, the most common QTLs are associated with body weight and feed conversion ratio.

## 4. Discussion

Improving our knowledge of the genetic structure in livestock populations is fundamental for improving selection designs and breeds, understanding environmental adaptation, enhancing the efficient use of the breeds, and implementing conservation programs. The advent of high-throughput genotyping arrays has considerably facilitated the study of genetic structure in local breeds, but they are infrequently used and generally understudied. This is the first study in which several Italian local poultry breeds from different locations (Figure 1) were characterized and compared through high-density genome-wide SNPs. Until now, different studies were limited to the regions of origin [17,18,19,20]. The high-resolution characterization of genomes increases knowledge on the genetic variability among these breeds. In this study, we investigated the patterns of genetic differentiation, diversity, and population structure among the populations, considered inside the TuBAvI project, to support the safeguarding of biodiversity and the genetic distinctiveness of local chicken breeds, as well as to improve the conservation programs [3,21].

In general, the levels of genetic diversity, investigated through different approaches, were the lowest in the local chicken breeds, including PPA, PPC, PPP, PRM, and SIC, compared to those in the commercial stocks. These results could be related to their reduced demographic sizes over time due to selection events. The average MAF values agree with the range reported for Dutch chickens, despite completely different histories and genetic backgrounds [22]. The observed and expected heterozygosity show different values compared to those from preview studies in the Veneto region based on microsatellite markers [3], as well as those for ANC, MOD, ROM, and VLD [23]. The results for BPT, BSA, PLN, PLB, MER, and SIC agree with previous studies [18]. There are no data in the literature on genetic variability of VLP, MUG, and COR. The local breeds compared with the commercial ones maintain lower observed and expected heterozygosity as expected [24]. The differences in the poultry breeds could be explained by an increase in inbreeding, linked to a reduction over time in the number of individuals within the breeds. Finally, the inbreeding coefficient (FHOM) shows low values in the commercial stocks, whereas the coefficient reaches high levels in the local chicken breeds, as expected, since these breeds have low numbers of individuals per population and undergo conservation schemes aiming to safeguard the populations. BSA is the only indigenous chicken breed showing a lower inbreeding level, probably due to its larger population compared to that of the others [25].

The genetic distances and relationships, as well as the population structure, were investigated through MDS, ADMIXTURE analysis, and Reynold’s genetic distances (Figure 2, Figure 3 and Figure 4). The MDS grossly separated the Italian local chicken breeds according to their genetic origin and/or the geographical proximity between breeding areas. The presence of a general North–South distribution of the genetic relationships in the Italian Peninsula was confirmed by the low genetic differentiation among some local breeds from the same geographic area, such as between the populations belonging to the Polverara and Padovana breeds, or among the three Sicilian breeds. Previously, a similar geographical pattern was described in other livestock species, such as Italian cattle [26], goat [27], and sheep breeds [28].

The results, from different analyses, agree with previous studies and with the breeding history of the chicken breeds under investigation [3,23]. The Padovana and Polverara breeds have close genetic relationships, as expected, as Padovana contributed to the origin of Polverara [3]. PLB, PLN, and MOD appear in a close neighborhood because they share the same historic crossbreeding [18,23]. In the same cluster, the ROM and ANC breeds are also present, as the same genetic background is maintained among the breeds as previously reported by other authors [23]. The VLD breed is closest to BPT and BSA, which show the same genetic background, as described in the literature [18]. We found a specific cluster for Sicilian breeds that includes SIC, COR, and VLP. These three breeds show a genetic identity related to their historical local origins, although their gene pools are affected by breeding with other breeds from other parts of the world. For instance, the SIC breed derives from the ancient inter-breeding of local Sicilian chickens with North African stocks [29]. PPP and MER belong to the same macro group in the MDS analysis; however, their genetic structure and identity are significantly different, even if they are dwarf breeds [7,30]. Finally, the commercial stocks show a close genetic identity with the PER, PRL, and PRM breeds, as confirmed by MDS and phylogenetic analysis; however, the genetic structure allows the discrimination of a subgroup in which only PER has the same genetic background as the commercial breeds (Figure 3, from *K* = 2 to 6). This could be because PER is a dual-purpose chicken, excellent both for egg and meat production. It has been crossed, as has its ancestors, to obtain commercial hybrids. In addition, the PER, PRL, and PRM breeds, as demonstrated by previous studies, shared a proportion of their common Anglo-American derivation [31].

Genome-wide SNPs are a powerful approach for detecting genomic regions with reduced homozygosity. ROH-based F estimates (*F_ROH_*) are increasingly used as an index of inbreeding or a signature of directional selection [22]. The genomic location of ROHs and their length are important genomic footprints of information on the demographic history of livestock species [32]. ROH analysis contains important information for conservation programs; within breeds, animals that show high levels of *F_ROH_*, as observed in the SIC, PPA, and PPP breeds, can be excluded for mating purposes in endangered populations to minimize the loss of genetic diversity and increase the biodiversity [22]. Commercial breeds show low levels of inbreeding and consequently have high genetic variability as expected. The analysis of ROH supports the genetic diversity estimates, underlining the main role of historical inbreeding in the genome of the local poultry breeds [22]. This aspect is related to recombination events, as a result of recent inbreeding traits, that compromise the integrity of long chromosome segments (long ROH over 10 Mb); conversely, shorter ROHs (about 1 Mb) indicate a more distant ancestral effect related to breed founder effects [33]. Thus, the MOD, PPA, SIC, and VLP breeds show a lower effect of recombination associated with recent evolutionary history, whereas PER, PPN, PPP, PRL, and PRM exhibit an older genetic identity compared to that of the other breeds.

After the identification of ROH islands on chromosomes, the presence of genes was observed. Several uncharacterized genes were revealed, reflecting the selection action on uncharacterized regulatory regions or simply the fixation of non-coding DNA by genetic drift due to uncontrolled selection [34]. In contrast, several coding genes are located inside the ROH islands. Among these genes, several are worth mentioning because they show a relation to specific traits related to livestock. *TPK1* (*thiamin pyrophosphokinase 1*) is the most important gene found on chromosome 2 and is involved in step 1 of the sub-pathway that synthesizes thiamine diphosphate from thiamine. This gene is conserved among several species and seems to be correlated with feed conversion and body weight [35]. On chromosome 5, *ANO* (*Anoctamin*) genes are involved in muscle tissue development and estrogen production [36]. Other candidate genes are *NELL1* (*NEL-like protein 1*), *SLC6A5* (*Solute carrier family 6 member 5*), and *BBOX1* (*gamma-butyrobetaine hydroxylase 1*), involved in growth factors, embryogenesis events, digestive enzyme activity, and feed efficiency, respectively [37,38,39]. The most representative gene on chromosome 7, with regards to production traits, is *FTCD*, which plays a main role in feed intake, in accordance with the quantitative trait loci identified [40]. Inside the last region identified on chromosome 8, the *TRMT1L* (*tRNA Methyltransferase 1-Like*) gene is noteworthy as it seems to be involved in chicken adaptation and survival in stressful conditions [41].

## 5. Conclusions

The TuBAvI project allowed us to evaluate the national poultry biodiversity of the Italian local breeds for the first time through genomic technologies that highlighted the heterogenic conservation of these populations. The results showed the existence of genetic variability and low inbreeding in almost all the breeds. The population structure and genetic distances show a clear separation among the breeds, with some particular clusters related to the region of origin. The genetic background of the commercial stocks is close to that of several breeds from the Veneto region, highlighting the story of their introgression with local breeds.

Through the present study, we provided a complete overview of the Italian chicken breeds and contextualized them at a national level. This will promote conservation plans and highlight their role as a genetic reservoir. Consequently, a strategy to increase value for these breeds should be provided in order to guarantee a profit for farmers. This could lead to the development of niche markets, representing the safest long-term strategy for local breed conservation.

This work will help with the design of targeted conservation plans. The information obtained represents a useful tool for understanding correct genetic management and supervising conservation activity. In this context, the information from genomic analysis may play a crucial role in the development of mating plans to avoid the negative effects of inbreeding in these breeds. Minor relatedness and low inbreeding are essential for small, local breeds to maintain the native genetic diversity, and good inbreeding management for the progeny is important for preserving biodiversity.

## Figures and Tables

**Table 1:** Genetic diversity indices. Number of animals per breed (N), minor allele frequency (MAF), expected (He) and observed (Ho) heterozygosity, and inbreeding coefficient (*F*_HOM_). For each value, the standard deviation (SD) is reported.

Breed	Acronym	N	MAF		Ho		He		*F*_HOM_	
			Mean	SD	Mean	SD	Mean	SD	Mean	SD
Ancona	ANC	24	0.267	0.242	0.263	0.181	0.274	0.187	0.284	0.100
Bianca di Saluzzo	BSA	24	0.286	0.190	0.339	0.172	0.336	0.151	0.076	0.059
Bionda Piemontese	BPT	22	0.283	0.210	0.325	0.186	0.317	0.164	0.116	0.025
Cornuta Caltanissetta	COR	22	0.267	0.301	0.167	0.162	0.210	0.178	0.545	0.180
Ermellinata di Rovigo	PER	23	0.309	0.321	0.199	0.192	0.220	0.198	0.459	0.044
Livorno Bianca	PLB	24	0.269	0.295	0.205	0.196	0.218	0.186	0.465	0.061
Livorno Nera	PLN	24	0.263	0.279	0.233	0.211	0.231	0.195	0.365	0.062
Mericanel della Brianza	MER	24	0.282	0.268	0.232	0.180	0.261	0.186	0.368	0.127
Millefiori di Lonigo	PML	23	0.281	0.238	0.293	0.199	0.291	0.178	0.202	0.080
Modenese	MOD	24	0.273	0.252	0.260	0.197	0.27	0.181	0.296	0.083
Mugellese	MUG	24	0.284	0.231	0.281	0.182	0.300	0.175	0.236	0.115
Padovana Argenta	PPA	24	0.241	0.331	0.151	0.198	0.146	0.185	0.588	0.098
Padovana Camosciata	PPC	24	0.238	0.303	0.169	0.191	0.179	0.193	0.538	0.095
Padovana Dorata	PPD	24	0.247	0.264	0.219	0.194	0.232	0.187	0.404	0.081
Pepoi	PPP	24	0.277	0.341	0.154	0.191	0.168	0.196	0.579	0.039
Polverara Bianca	PPB	24	0.260	0.261	0.216	0.179	0.248	0.187	0.411	0.052
Polverara Nera	PPN	24	0.257	0.290	0.201	0.193	0.213	0.194	0.454	0.062
Robusta Lionata	PRL	23	0.305	0.345	0.181	0.199	0.185	0.195	0.508	0.039
Robusta Maculata	PRM	24	0.304	0.358	0.157	0.190	0.166	0.193	0.572	0.032
Romagnola	ROM	24	0.271	0.241	0.281	0.197	0.278	0.182	0.235	0.091
Siciliana	SIC	24	0.259	0.361	0.129	0.205	0.123	0.189	0.648	0.034
Valdarnese	VLD	24	0.283	0.204	0.321	0.181	0.322	0.160	0.127	0.098
Valplatani	VLP	20	0.281	0.268	0.280	0.224	0.261	0.184	0.239	0.086
708 Broiler Ross	708	13	0.317	0.234	0.369	0.219	0.324	0.162	−0.005	0.009
Eureka	EUK	9	0.329	0.261	0.374	0.260	0.305	0.177	−0.018	0.013
Hy-lyne white eggs	HYL	10	0.333	0.278	0.375	0.286	0.289	0.285	−0.020	0.008
Isa Brown	ISA	9	0.332	0.261	0.378	0.276	0.298	0.182	−0.028	0.017

**Table 2:** Statistical indices for runs of homozygosity in the analyzed Italian breeds: F_ROH_, mean runs of homozygosity (ROH) based on inbreeding coefficient with standard deviation; mean ROH, mean number of ROH per individual and per breed with standard deviation; total number of ROH per breed.

Breed	F_ROH_	SD	Mean ROH	SD	Total Number ROH
Ancona (ANC)	0.201	0.099	56.21	14.01	1351
Bianca di Saluzzo (BSA)	0.081	0.057	20.53	9.22	492
Bionda Piemontese (BPT)	0.081	0.024	31.52	6.82	694
Cornuta di Caltanissetta (COR)	0.507	0.184	80.01	29.72	1761
Ermellinata di Rovigo (PER)	0.305	0.082	133.71	24.83	3077
Livorno Bianca (PLB)	0.427	0.059	77.72	6.15	1865
Livorno Nera (PLN)	0.296	0.063	68.18	7.94	1636
Mericanel della Brianza (MER)	0.326	0.135	65.15	15.97	1563
Millefiori di Lonigo (PML)	0.166	0.073	56.01	20.79	1289
Modenese (MOD)	0.264	0.086	54.14	9.42	1299
Mugellese (MUG)	0.225	0.112	39.64	16.29	951
Padovana Argentata (PPA)	0.509	0.118	96.76	12.71	2323
Padovana Camosciata (PPC)	0.410	0.109	103.52	17.74	2485
Padovana Dorata (PPD)	0.230	0.070	100.42	20.66	2410
Pepoi (PPP)	0.482	0.096	151.81	30.76	3645
Polverara Bianca (PPB)	0.310	0.068	113.85	22.07	2732
Polverara Nera (PPN)	0.353	0.087	127.80	21.91	3069
Robusta Lionata (PRL)	0.353	0.109	135.11	26.44	3109
Robusta Maculata (PRM)	0.410	0.113	157.58	22.06	3782
Romagnola (ROM)	0.187	0.091	43.17	10.04	1054
Siciliana (SIC)	0.607	0.037	96.09	6.90	2305
Valdarnese (VLD)	0.121	0.095	30.76	15.60	737
Valplatani (VLP)	0.236	0.087	41.55	5.91	830
708 Broiler ROSS (708)	0.034	0.009	17.24	4.31	224
Eureka (EUK)	0.033	0.005	17.74	2.74	160
Hy-lyne white eggs (HYL)	0.038	0.008	19.23	3.59	192
IsaBrown (ISA)	0.030	0.011	16.22	5.95	146

**Table 3:** ROH islands identified in Italian chicken breeds represent the genomic regions of extended homozygosity. *Gallus gallus* chromosome number (GGA), number of SNP per region (no. of SNPs), start and end points (Start/End), length of region (length in bp), genes inside the islands (Genes), and quantitative trait loci (QTLs) are reported.

GGA	No. of SNPs	Start	End	Length (bp)	Genes	QTL
2	18	53,138,767	53,202,574	63,807	*TPK1, LOC107051643*	-
5	315	2,124,338	3,730,724	1,606,386	*NELL1, SLC6A5, LOC107053351, LOC107053349, LOC107053350, LOC107053348, ANO5, SLC17A6, FANCF, GAS2, SVIP, ANO3, SLC5A12, BBOX1, SLC5A12, FIBIN, CCDC34, LGR4, LIN7B*	Body weight (28 days) QTL (95,416) Body weight (28 days) QTL (95,415)
7	273	6,771,434	7,892,629	1,121,195	*COL6A2, LOC107053768, LOC107053769, LOC107053763, FTCD, MCM3AP, YBEY, LOC107053762, MCM3AP, YBEY, POFUT2, LOC107053766, CD163L1, LSS, S100B, DIP2A, PCNT, KMO, FAM207a, ITGB3, ADARB1*	Feed conversion ratio QTL (139,597) Feed conversion ratio QTL (139,472) Feed conversion ratio QTL (139,435) Feed conversion ratio QTL (139,598)
8	371	9,506,680	10,604,288	1,097,608	*LOC101751732, PLA2G4A, PTGS2, PDC, C8H10RF27, TPR, LOC100859371, HMCN1, LOC107053953, LOC101750397, LOC107053952, INVS1ABP, SWT1, TRMT1L, LOC107053951*	Feed conversion ratio QTL (139,596)

## Supplementary Materials

The following are available online at https://www.mdpi.com/2076-2615/10/8/1441/s1. Figure S1. Seven categories used for the classification of runs of homozygosity (ROH), according to size (from <5 to more than 30 Mb). On the y-axis, the mean sum of ROH is in Mb, whereas on the x-axis is each ROH length category per breed.
