# Supplementary figures and images for: Genome-Wide SNP Analysis Reveals the Population Structure and the Conservation Status of 23 Italian Chicken Breeds
Source: Animals (Basel). 2020 Aug 18;10(8):1441. doi: 10.3390/ani10081441 (PMC7460279; doi:10.3390/ani10081441)

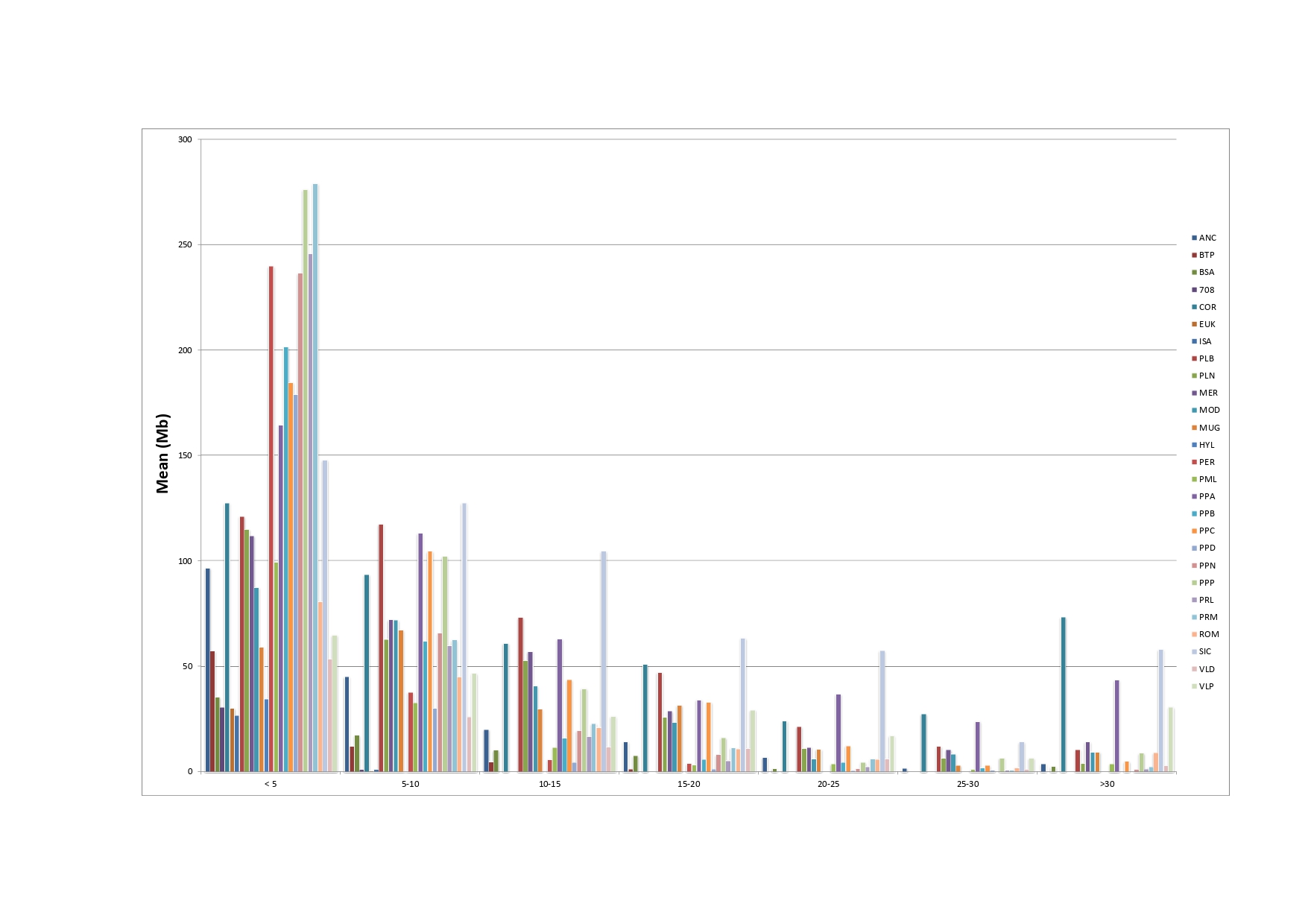

Supplement: Supplementary file 1 [file animals-10-01441-s001.zip › Figure S1.jpg]
